# Investigation of *Lonicera japonica* Flos against Nonalcoholic Fatty Liver Disease Using Network Integration and Experimental Validation

**DOI:** 10.3390/medicina58091176

**Published:** 2022-08-30

**Authors:** Chun-Yong Sun, Pan Zhao, Pei-Zheng Yan, Jia Li, Dong-Sheng Zhao

**Affiliations:** College of Pharmacy, Shandong University of Traditional Chinese Medicine, Jinan 250355, China

**Keywords:** *Lonicera japonica* Flos, nonalcoholic fatty liver disease, network integration, molecular docking, experimental pharmacology

## Abstract

*Background and objective*: *Lonicera japonica* Flos (LJF) is a well-known traditional herbal medicine that has been used as an anti-inflammatory, antibacterial, antiviral, and antipyretic agent. The potent anti-inflammatory and other ethnopharmacological uses of LJF make it a potential medicine for the treatment of nonalcoholic fatty liver disease (NAFLD). This research is to explore the mechanisms involved in the activity of LJF against NAFLD using network integration and experimental pharmacology. *Materials and methods*: The possible targets of LJF involved in its activity against NAFLD were predicted by matching the targets of the active components in LJF with those targets involved in NAFLD. The analysis of the enrichment of GO functional annotations and KEGG pathways using Metascape, followed by constructing the network of active components–targets–pathways using Cytoscape, were carried out to predict the targets. Molecular docking studies were performed to further support the involvement of these targets in the activity of LJF against NAFLD. The shortlisted targets were confirmed via in vitro studies in an NAFLD cell model. *Results*: A total of 17 active components in LJF and 29 targets related to NAFLD were predicted by network pharmacology. Molecular docking studies of the main components and the key targets showed that isochlorogenic acid B can stably bind to TNF-α and CASP3. In vitro studies have shown that LJF down-regulated the TNF-α and CASP3 expression in an NAFLD cell model. *Conclusions*: These results provide scientific evidence for further investigations into the role of LJF in the treatment of NAFLD.

## 1. Introduction

Nonalcoholic fatty liver disease (NAFLD) is defined as a condition in which fat accumulates in the liver instead of being caused by excessive alcohol consumption [1]. According to relevant investigation, the incidence of NAFLD in European and Asian countries is 14% to 21%, while in the United States it is 11% (male 14%, female 8%) [2]. The reasons for its occurrence are obesity, dyslipidemia, insulin resistance, and type 2 diabetes and other diseases, which mainly increase the fasting blood glucose, blood lipid levels, and blood pressure [3]. Several studies were carried out to understand the pathogenesis of NAFLD and possible therapeutic interventions [4]. However, the pathogenesis mechanisms of NAFLD are not yet fully understood, and some effective drug candidates to cure NAFLD are still in the pipeline [5]. Thus, it is necessary to conduct research to find appropriate medicines in treating NAFLD, and at present there is a lot of effective traditional Chinese medicine (TCM) available for the treatment of NAFLD [6,7].

*Lonicera japonica* Flos (LJF), a member of the Caprifoliaceae family, has a long history of use by humans as a functional food and a TCM [8]. It is most widely used as an anti-inflammatory, antibacterial, antibacterial, antiviral, antioxidant, antilipidemic, and antipyretic medicine [9]. The potent anti-inflammatory and other ethnopharmacological activities of LJF make it a potential medicine for the treatment of NAFLD. Currently, studies have shown that LJF is useful in protecting livers. Animal studies have shown that ethanol extracts of LJF reverse the methionine- and choline-deficient diet-fed induced nonalcoholic steatohepatitis. The ethanol extract of LJF contains chlorogenic acid (68.2 ± 0.3 µg/g) [10]. Another animal study has shown that LJF protects lives from carbon tetrachloride and alcohol injury [11,12]. Besides, LJF also has certain efficacies in the treatment of metabolic syndrome, such as hypertension, hyperglycemia, obesity, and hyperlipidemia. However, there are no reported studies on the effect of LJF against NAFLD.

In recent years, new technologies including network integration pharmacology have been developed to determine the activity and mechanisms of TCM. With this multidisciplinary research, it is possible to predict the active components of TCM and their potential biological targets. Additionally, the mechanisms of action of TCM and the interaction between the active compounds and biological targets can be explored by network pharmacology, which will be of great help in elucidating the pharmacological characteristics of TCM [13,14].

In this study, we have used the multiprong approach, which combines network integration, molecular docking studies, and in vitro studies to identify the bioactive compounds in LJF and their potential biological targets, signaling pathways, and molecular mechanisms in treating NAFLD.

## 2. Materials and Methods

### 2.1. Screening of Active Components in LJF

Li et al. has performed metabolomic studies on LJF in healthy rats and identified 25 compounds, out of which 15 are LJF compounds and the remaining 10 are their metabolites [15]. In this study, we have chosen the compounds with OB ≥ 30% and DL ≥ 0.18 as candidate components of LJF [16,17].

### 2.2. Target Prediction of Active Compounds in LJF

We used the PubChem (https://pubchem.ncbi.nlm.nih.gov/, accessed on 12 March 2020) database to convert all compounds into the standard Canonical SMILES format and obtained the 2D structure of compounds. Then, we imported the SMILES format file into Swiss Target Prediction (http://www.swisstargetprediction.ch/, accessed on 12 March 2020) platform to get potential targets in humans (Homo sapiens) [18].

### 2.3. Collection of Potential Targets of LJF against NAFLD

The genes involved in NAFLD were collected from three disease target databases; OMIM (http://www.omim.org/, accessed on 13 March 2020), GeneCards (https://www.genecards.org/, accessed on 13 March 2020) and NCBI gene (https://www.ncbi.nlm.nih.gov/gene/, accessed on 13 March 2020) [19,20]. The duplicate genes were removed, and the association between these genes and compounds of LJF were investigated. The targets involved in NAFLD, which are in common with the targets of active components of LJF, were selected as potential LJF targets for treating NAFLD.

### 2.4. Construction of Protein-Protein Interaction (PPI) Network for Potential Targets of LJF against NAFLD

We uploaded the potential targets of LJF against NAFLD to the large-scale STRING protein database (https://string-db.org/, accessed on 14 March 2020) and selected the race as “Home sapiens” to obtain PPI network.

### 2.5. GO Annotation and KEGG Pathways Enrichment Analysis

The Metascape platform (https://metascape.org/, accessed on 14 March 2020) is a gene annotation analysis database for enrichment analysis of GO annotation and KEGG pathways of input genes. The analysis is also useful to make new predictions based on the information in the existing databases [21]. The potential targets of LJF in treating NAFLD were uploaded into the Metascape platform and selected “H. sapiens” as the input and analysis species with a *p*-value < 0.01. Then GO and KEGG pathway enrichment analysis of potential targets were performed. The top ranked GO annotation and KEGG pathways were screened according to the number of targets contained in each entry, and the results were visualized using GraphPad Prism 7.0 software (create by Dotmatics, http://www.graphpad.com/, accessed on 14 March 2020).

### 2.6. Acquisition Protein Function Information for Potential Targets of LJF against NAFLD

Annotation analysis of genes in the Metascape platform includes enrichment of biological processes, pathways of input genes, the functions of the genes and target types associated with these genes. The above-mentioned analysis was carried out to obtain the information on the function of proteins.

### 2.7. Construction of “Active Ingredient–Target–Pathway” Network for LJF against NAFLD

The data of the active ingredients of LJF, their potential targets for treating NAFLD, and metabolic pathways were imported into Cytoscape 3.6.1 software to construct a network model of “active ingredient–target–pathway” for LJF treating NAFLD.

### 2.8. Molecular Docking Studies on the Key Active Ingredients with the Potential Targets

To validate the above results, molecular docking studies were carried out to calculate the binding affinity of the active ingredients toward the key hubs in the network. The active components with most targets and the proteins with high-degree nodes in the gene network were chosen to perform molecular docking studies. The 3-dimensional (3D) structure of ligand was downloaded from the PubChem database. A database (http://www.rcsb.org/, accessed on 15 April 2020) was used to obtain the 3D structures of the target receptor. The molecular docking process, including ligand and receptor preparation, was done using Surflex-Dock module of the Sybyl-X 2.1.1 program (Tripos, St. Louis, MO, USA) and ranked based on Surflex-Dock score (total score) [22]. The graphical visualization of molecular docking studies data was done with PyMOL 2.3 tool.

### 2.9. Lipid Accumulation Detection

LJF (20210710) was purchased from Pingyi county of Shandong province and was authenticated by Yong-Qing Zhang of Shandong University of Traditional Chinese Medicine. The material was powdered, passed through a 60-mesh sieve, and extracted with 65% (*v/v*) ethanol (1: 10 ratio) under reflux for 1 h and filtered. The filtered residue was again extracted under reflux with 65% *v/v* ethanol for 0.5 h and filtered. The filtrates were combined, concentrated under vacuum to distill off ethanol, and then lyophilized. The main 12 components of LJF extract were determined using high-performance liquid chromatography (HPLC) (Supplemental Figure S1). The components are neochlorogenic acid 0.98%, loganic acid 1.28%, chlorogenic acid 25.84%, cryptochlorogenic acid 1.27%, loganin 3.84%, secoxyloganin 3.72%, rutinum 1.11%, hyperoside 1.65%, isochlorogenic acid B 2.21%, isochlorogenic acid A 3.49%, isochlorogenic acid C 11.54%, and luteoloside 2.89%.

HepG2 cells were cultured in the incubator (37 °C and 5% CO_2_) with DMEM complete medium containing 10% fetal bovine serum, 100 μg·mL^−1^ penicillin, and 100 μg·mL^−1^ streptomycin. HepG2 cells in the logarithmic growth phase were chosen for the following experiments. HepG2 cells were seeded in a 96-well plate at a cell density of 2 × 10^4^ cells/mL and cultured in an incubator for 12 h. HepG2 cells were divided into three groups: (1) control group in which cells were treated with DMEM complete medium, (2) NAFLD model group in which cells were treated with a complete medium containing 200 μmol·L^−1^ sodium oleate, and (3) LJF group in which cells were treated with a complete medium containing 200 μmol·L^−1^ sodium oleate and 25 mg/L LJF extract. The three cell groups (control, model, and LJF) were stained with oil red O stain to investigate the accumulation of lipids.

### 2.10. Detection of ALT and AST Content in HepG2 Cells

After the three cell groups (control, model, and LJF) were cultured for 24 h, the cells were collected and homogenized. The alanine transaminase (ALT) and aspartate aminotransferase (AST) content in HepG2 cells was determined using Alanine Transaminase Activity Assay Kit and Aspartate Aminotransferase Activity Assay Kit (Nanjing Jiancheng Bioengineering Institute, Nanjing, China), respectively.

### 2.11. TNF-α and CASP3 Expressions Were Detected in HepG2 Cells

The qRT-PCR assay was used to detect tumor necrosis factor (TNF-α) and caspase 3 (CASP3) mRNA levels in three cell groups (control, model, and LJF). The HepG2 cells from three groups were seeded in a 60 mm diameter Petri dish at a cell density of 2 × 10^6^ cells/mL. After 24 h, the RNA was extracted with a total RNA extraction kit. The purity of mRNA was determined using electrophoresis using agarose gel and TAE buffer. The mRNA was reverse-transcribed to cDNA using the Hiscript II 1st Strand cDNA Synthesis Kit. Finally, QuantiNovaTM SYBR Green PCR Kit was used for quantitative estimation of the genes. The primer sequences of TNF-α and CASP3 used in this study are shown in Supplementary Table S1.

Western blot (WB) assay was used to detect TNF-α and CASP3 protein levels in all three cell groups (control, model, and LJF). Cells in three groups were seeded in a 96-well plate at a cell density of 2 × 10^4^ cells/mL and cultured for 24 h. Then, the culture medium was discarded, and 3.7% formaldehyde was added to fix the cells for 20 min. Then, the formaldehyde was replaced with 0.1% Triton, and the plate was shaken slowly for 5 min to permeabilize the cells. The above step was repeated five times. Skimmed milk (5%) was added to the wells and incubated for 1.5 h, followed by incubation with the primary antibody diluted with 5% skimmed milk (1:1000; BOSTER Biological Technology co. Ltd., Beijing, China) at 4 °C for 16 h. Then, the cells were incubated with 5% fluorescent secondary antibody (1:1000; BOSTER Biological Technology Co., Ltd., Beijing, China) diluted with skim milk at 37 °C for 1 h in the dark. Imaging and analysis were performed under the ODYSSEY CLx dual infrared laser imaging system.

### 2.12. Statistical Analysis

SPSS 26.0 software was used for statistical analysis of the data, and the data were expressed as mean ± SD. One-way analysis of variance was used to compare the statistical differences between the groups. *p* < 0.05 is considered statistically significant, and *p* < 0.01 is considered very significant.

## 3. Results

### 3.1. Screening of LJF Active Components

We have selected 17 compounds with Oral Bioavailability (OB) ≥30% and Drug Likeness (DL) ≥0.18, via the study of Li et al. [15]. The names of the compounds, their chemical formulae, and 2D structures are shown in Table 1.

### 3.2. Prediction of Target Genes

**Figure 1:**
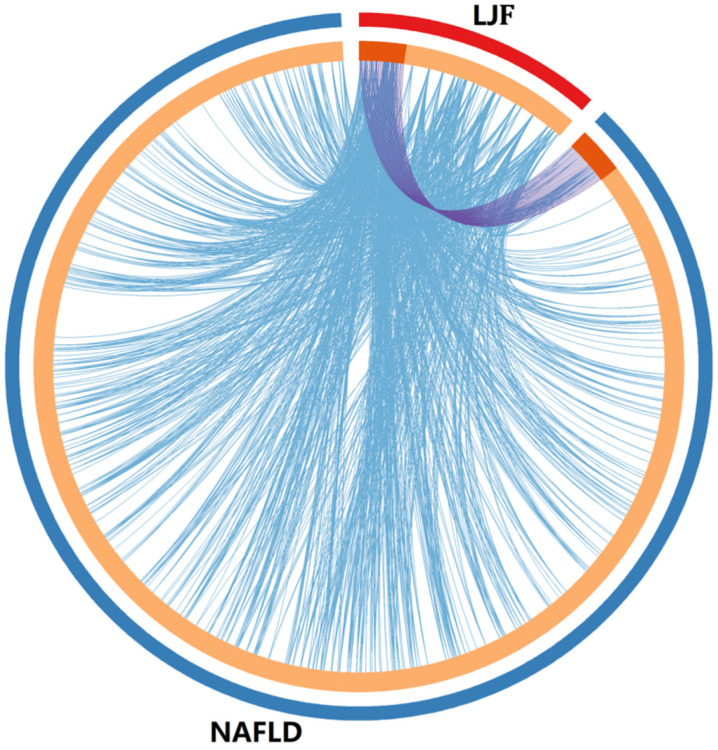
Overlap between LJF target genes and NAFLD-related target genes. (1) Only at the gene level, where purple curves link identical genes; (2) including the shared term level, where blue curves link genes that belong to the same enriched ontology term. The inner circle represents gene lists, where hits are arranged along the arc. Genes that hit both LJF target genes and NAFLD-related target genes lists are colored in dark orange, and genes unique to a list are shown in light orange.

The Swiss Target Prediction platform was used to predict the potential targets of the active compounds, and a total of 142 potential target genes were identified after removal of the duplicates. By comparing these target genes with NAFLD-related genes from the OMIM, Gene Cards, and NCBI gene databases, via the Metascape platform, the intersection of the genes were shortlisted in Figure 1. Supplementary Table S2 shows that the shortlisted 29 potential targets play a role in the treatment of NAFLD by LJF.

### 3.3. PPI Network for Potential Targets of LJF against NAFLD

**Figure 2:**
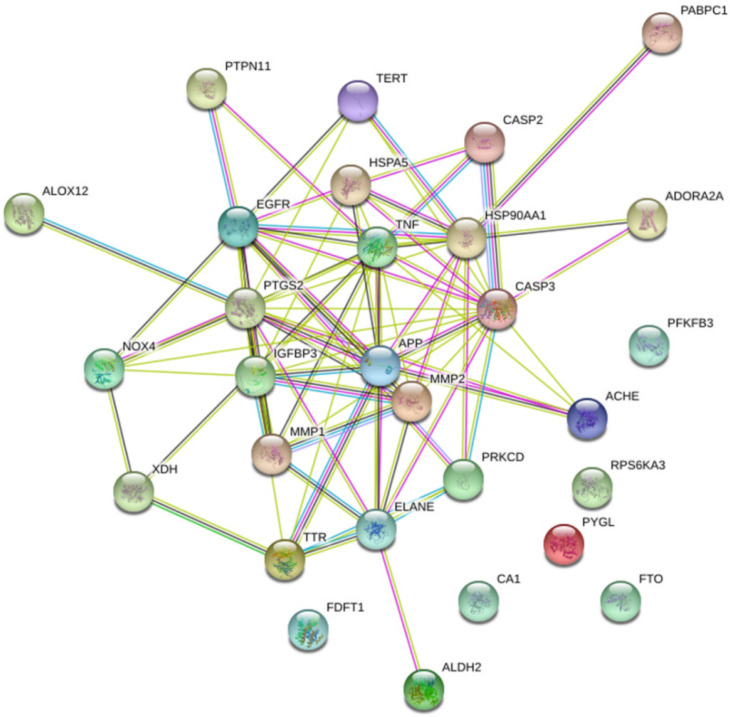
PPI Network for potential targets of LJF against NAFLD. Network nodes represent proteins; edges represent protein–protein associations, blue edges represent known interactions from curated databases, purple edges represent known interactions experimentally determined, yellow edges represent interactions from text mining.

To explore the possible mechanism of action of LJF in treating NAFLD, the 29 potential target proteins were input into the STRING database to construct a PPI network of target protein interactions (Figure 2). The network has 29 nodes and 79 interactions, with an average degree of 5.45, of which six target proteins, including CA1, PYGL, FTO, PRS6KA3, FDFT1, and PFKFB3, have no interaction with other proteins. The identified top-ranked target proteins have shown diverse and beneficial functions for treating NAFLD. The degree of the nodes is high, and there are some nodes with a larger number of edges in the network, such as TNF with 17 edges, CASP3 with 16 edges, PTGS2 and EGFR with 13 edges, IGFBP3 and APP with 10 edges, ELANE with 9 edges, MMP2 with 8 edges, MMP1 and HSPA5 with 7 edges, and TTR with 6 edges. These targets were found to be firmly associated with other targets in the network and, therefore, might play a vital role for LJF in treating NAFLD.

### 3.4. GO Annotation and KEGG Pathways Enrichment Analysis

**Figure 3:**
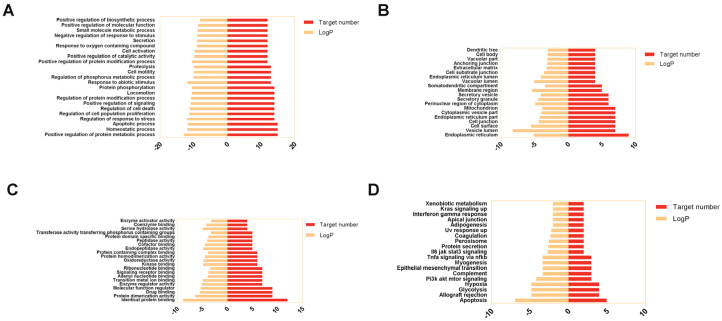
Enrichment analysis of potential targets from active ingredients of LJF against NAFLD. Analysis of Gene Ontology terms for biological process (**A**), cellular component (**B**) and molecular function (**C**); Analysis of KEGG pathways (**D**). Bars colored in dark orange represent the number of targets involved in each entry; Bars colored in light orange represent log p.

The Metascape database was used to perform GO and KEGG pathway enrichment analysis on 29 potential targets for LJF in treating NAFLD. We have set the threshold *p*- value < 0.01 and screened the top GO annotation and KEGG pathways. GraphPad Prism 7.0 was used to visualize the results (Figure 3). GO annotation analysis is referred to as an item that describes the biological function of a gene through the same semantics, which consists of biological process (BP), cellular component (CC), and molecular function (MF).

The 23 top-ranked terms of GO BP are mainly involved in the metabolic process, homeostatic process, apoptotic process, response to stress, cell proliferation, cell death, signaling, protein modification process, locomotion, protein phosphorylation, response to abiotic stimulus, phosphorus metabolic process, cell motility, proteolysis, positive catalytic activity, cell activation, response to oxygen-containing compounds, and secretion (Figure 3A).

The 20 top-ranked entries of GO CC were enriched in the endoplasmic reticulum, vesicle lumen, cell surface, cell junction, endoplasmic reticulum, cytoplasmic vesicles, mitochondrion, perinuclear region of cytoplasm, secretory granule, secretory vesicle, membrane region, somatodendritic compartment, vacuolar lumen, endoplasmic reticulum lumen, cell-substrate junction, extracellular matrix, anchoring junction, vacuolar part, cell body, and dendritic tree (Figure 3B).

The 21 top-ranked terms of GO MF are involved in protein binding, protein dimerization activity, drug binding, molecular function regulation, enzyme regulation, transition metal ion binding, adenyl nucleotide binding, signaling receptor binding, ribonucleotide binding, kinase binding, oxidoreductase activity, protein homodimerization, protein containing complex binding, endopeptidase activity, cofactor binding, peptidase activity, protein domain specific binding, transferase activity transferring phosphorus containing groups, serine hydrolase activity, coenzyme binding, and enzyme activator activity (Figure 3C).

A total of 19 pathways were associated with LJF against NAFLD through the KEGG pathway enrichment analysis in the Metascape database (Figure 3D). The pathways are apoptosis, allograft rejection, glycolysis, hypoxia, PI3K-AKT-mTOR signaling, complement, epithelial-mesenchymal transition, myogenesis, TNF signaling via NFkB, IL6-JAK-STAT3 signaling, protein secretion, peroxisome, coagulation, UV response up, adipogenesis, apical junction, interferon-gamma response, Kras signaling, and xenobiotic metabolism.

### 3.5. Acquisition Protein Function Information for Potential Targets of LJF against NAFLD

The 29 top-ranked potential targets were imported into the Metascape database, and the functions corresponding to the targets were obtained. The results (Supplemental Table S3) showed that transcription factors, receptors, enzymes, and ion channels are mainly involved in the possible activity of LJF against NAFLD. 

### 3.6. Construction of “Active Ingredient-Target-Pathway” Network for LJF against NAFLD

**Figure 4:**
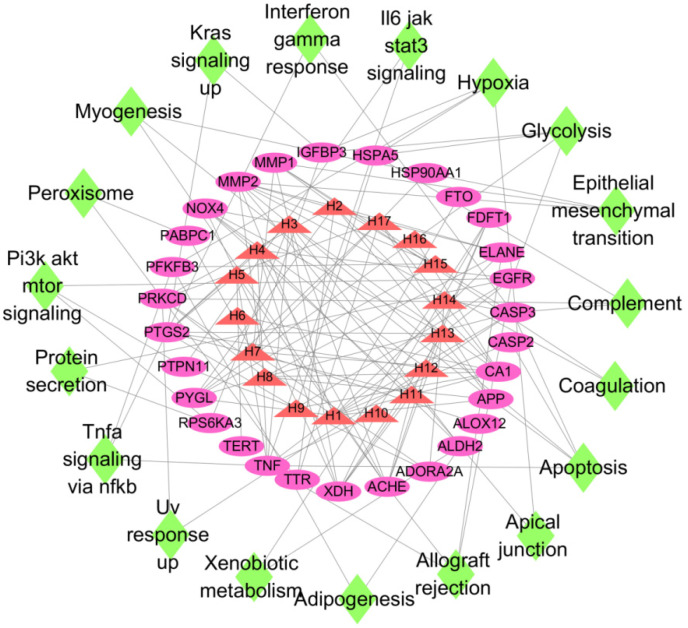
Components–targets–pathways network of LJF against NAFLD. Nodes with different colors represent active ingredients, targets, and pathways. Red triangle nodes represent active ingredients in LJT, purple oval nodes represent targets, and green diamond nodes represent pathways.

The information on active ingredients, targets, and KEGG pathways of LJF were imported into Cytoscape software to build an “active ingredient–target–pathway” network. In the output network (Figure 4), nodes with different colors represent active ingredients, targets, and pathways. Red triangle nodes represent active ingredients in LJF, purple oval nodes represent targets, and green diamond nodes represent pathways. If a target is a potential target of an active compound, they are connected by edges. If a protein has a regulatory effect on a pathway, they are also joined by edges. As shown in Figure 4, numerous targets are associated with one active component, and numerous components are associated with one target. This type of association reflects that LJF may be useful in treating NAFLD, and the activity is due to multiple components mediated through multiple targets and multiple pathways.

### 3.7. Molecular Docking Validation of the Key Ingredients and Target Proteins

**Figure 5:**
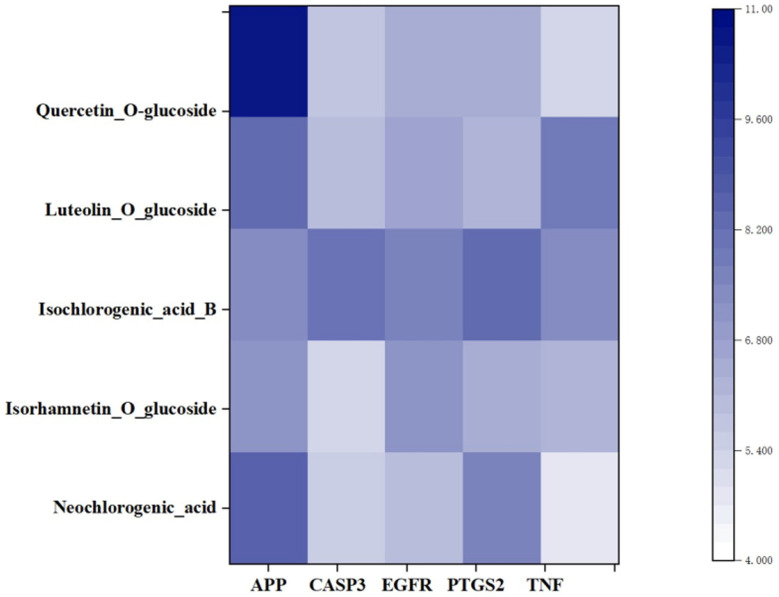
Heatmaps shows Surflex-Dock score (total score) of top 5 hub genes combining to 5 key bioactive compounds in LJF.

**Figure 6:**
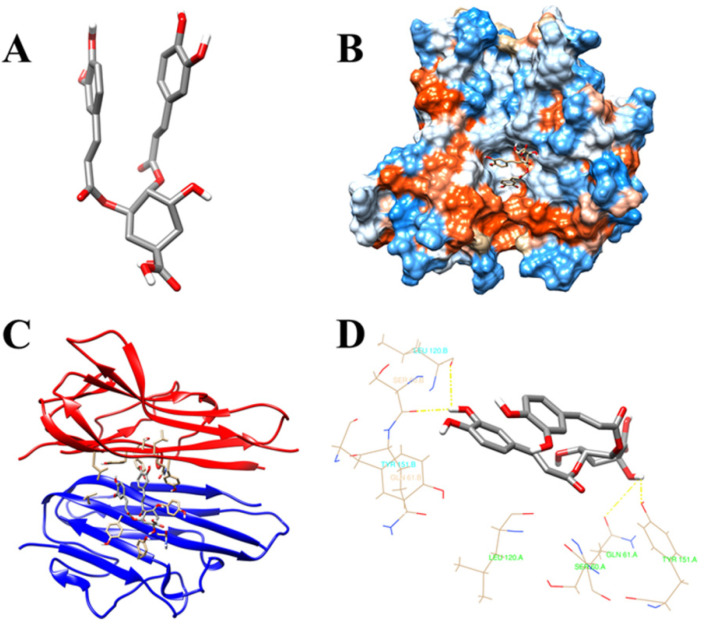
Binding mode of isochlorogenic acid B with TNF. The 3D structure of isochlorogenic acid B (**A**) and the 3D crystal structure of human TNF (**B**). Isochlorogenic acid B stably dock with the endogenous ligand binding site in TNF (**C**) and the potential interactions between isochlorogenic acid B with TNF (**D**).

Molecular docking has been widely used to verify the affinity between the key active substances and hub target proteins. To display the docking results, we have selected the five top-ranked genes and compounds in the network pharmacology, as shown in the heatmap (Figure 5). As shown in the heatmap, the five key compounds in LJF have a very certain binding affinity to the 5 top-ranked genes, especially, as 70% of them reveal stronger binding affinity. In this study, isochlorogenic acid B with 44 edges and TNF with 17 edges in the gene network were selected as the key active substance and hub target protein, respectively. The 3D structure of isochlorogenic acid B (Compound CID: 5281780) was downloaded from the PubChem database (Figure 6A). The 3D structure of TNF was obtained from the protein database http://www.rcsb.org/ (accessed on 15 April 2020) (Figure 6B). Docking studies have successfully predicted the docking poses and affinity between isochlorogenic acid B and the binding site of TNF (Figure 6C). The total binding score of the key compound, isochlorogenic acid B, with TNF was found to be 7.3706, suggesting stronger affinity. During the binding process, isochlorogenic acid B was bound stably with TNF and amino acid residues, such as LEU120, SER60, GLN61, and TYR151, were involved in the binding (Figure 6D). 

### 3.8. TNF-α and CASP3 Expression Were down Regulated in LJF-Treated NAFLD Model Cells

**Figure 7:**
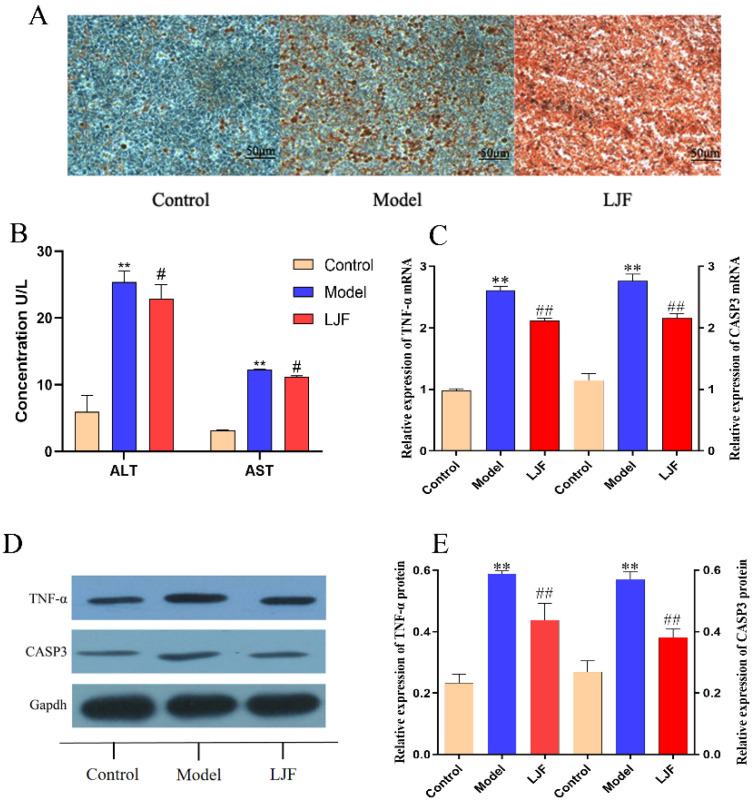
(**A**) The effect of LJF in HepG2 cells detected by oil red O staining. (**B**) The effect of LJF on the content of ALT and AST in HepG2 cells. (**C**) The effect of LJF on the expression of TNF-α and CAPS3 mRNA in HepG2 cells. (**D**,**E**) The effect of LJF on the expression of TNF-α and CAPS3 protein in HepG2 cells. **^,^ p < 0.01; *^#,^ p* < 0.05; *^##,^ p* < 0.01. ALT, alanine transaminase; AST, aspartate aminotransferase; TNF-α, tumor necrosis factor; CASP3, caspase 3.

HepG2 cells were stimulated with sodium oleate (200 μmol·L^−1^) to develop a lipid accumulation model, and oil red O staining was used to detect the lipid content. Figure 7A showed that the oil red O level in the cells of the model group was significantly higher than that of the control group, which indicated that the NAFLD cell model was successfully established. Compared with the model group, LJF extract down-regulated the intracellular lipid level, as shown in Figure 7A. Compared with the control group, the ALT and AST levels in the model group were significantly elevated (*p* < 0.01). Compared with the model group, the ALT and AST levels in the LJF group were significantly decreased (*p* < 0.05) (Figure 7B). 

To further explore the mechanism of LJF in treating NAFLD, the real-time qPCR and Western blot methods were used to quantify the mRNA and protein expression of TNF-α and CAPS3. Compared with the control group, sodium oleate has significantly up-regulated the mRNA and protein expression of TNF-α and CAPS3 (*p* < 0.01) (Figure 7C–E). LJF extract has down-regulated the mRNA and protein expression of TNF-α and CAPS3 (*p* < 0.01), as shown in Figure 7C–E. From these results, it is suggested that TNF-α and CAPS3 might be the potential targets for LJF in treating NAFLD.

## 4. Discussion

TCM plays a unique and essential role in alternative medicine, the pharmaceutical industry, and in many other fields [23,24]. Nonetheless, TCM also faces many challenges in terms of quality control, elucidation of pharmacological actions, complex chemistry, and complex mechanisms of action [25]. The main principle of TCM treatment is curing diseases at the root level and rehabilitating biological functions of the organs, and, thus, the concept in TCM is comparable to the concept of network pharmacology [26].

There are significant obstacles facing traditional research methods for TCM, including long cycles, low data flux, low precision, and high costs. Moreover, it is difficult to elucidate the pharmacological actions of TCM and their mechanisms by conventional research methods. The network pharmacology, target predictions, and molecular docking studies have provided new avenues of research for exploring the pharmacological actions and mechanisms of TCM [27]. TCM has been playing a vital role in the treatment of NAFLD and has shown significant beneficial effects [28]. 

In this study, we have conducted an integrated approach, which combines network pharmacology and molecular docking approaches to predict 17 active ingredients in LJF that exhibit activity on 29 potential targets relevant to NAFLD. This approach can be considered as multi-component–multi-target–multi-pathway process to unveil the involvement of multiple proteins, multiple pathways, and multiple biological processes. In vitro studies revealed that LJF extract can downregulate TNF-α and CASP3 expressions in an NAFL cell model, which further confirmed the mechanism of LJF against NAFLD. 

A study has demonstrated that TNF-α plays an essential part in the development of NAFLD by upregulating the mediators of fibrosis and lipid metabolism and upregulating the inflammatory cytokines in the liver [29]. Caspases are a family of intracellular cysteine proteases that mediate inflammation and apoptosis through activating pro-inflammatory cytokines, such as IL-1β, IL-18, and IL-33 [30]. Activation of caspase-3 (CASP3) is identified as one of the critical pathways in liver injury, resulting in fibrosis, inflammation, and hepatocyte apoptosis [31,32]. It is reported that the cyclooxygenase -2 (PTGS2) selective inhibitor suppresses the development of NAFLD in a diet-induced obesity rat model [33]. Another study has reported that an EGFR inhibitor (erlotinib) reduces the number of activated HSCs by depressing EGFR phosphorylation [34]. 

IGFBP-3 is reported to exhibit anti-inflammatory activity in hepatocytes by downregulating the production of proinflammatory cytokines through the NF-κB and JNK pathways. The secretion of IGFBP-3 is inhibited under lipotoxic conditions, boosting palmitate-induced IL-8 synthesis and secretion [35]. 

Amyloid precursor protein/presenilin-1 (APP/PS1) transgenic mice fed with a cholate, cholesterol, and high-fat diet is reported to induce NAFLD without hyperglycemia and obesity. NAFLD leads to cognitive decline and cerebral hypoperfusion in APP/PS1 mice [36]. A recent study has reported the presence of MMP-1 positive HPCs in the early stage of NAFLD [37].

Recent study has shown that apoptosis is the principal mechanism contributing to hepatocellular death in NAFLD [38]. Another study in animals has demonstrated that liver fibrosis is mediated through liver tissue hypoxia in hepatic steatosis via hepatocyte HIF 1 [39]. Several studies have reported that the abnormal autophagy plays a crucial role in the pathogenesis of NAFLD [40]. Acetylshikonin is reported to ease NAFLD by raising the hepatocyte autophagy via the mTOR pathway [41]. The serum complement C3 levels are reported to be associated with the severity, risk, and prevalence of NAFLD but not with metabolic syndrome and obesity [42]. Another study has shown that hedgehog-mediated epithelial–mesenchymal transition in ductular cells plays a role in the pathogenesis of liver cirrhosis in NAFLD [43]. The increase in relative skeletal muscle mass has beneficial effect in reversing and preventing NAFLD [44].

## 5. Conclusions

In this study, the network pharmacology was integrated with molecular docking studies to predict the underlying mechanism of LJF in the treatment of NAFLD. We have found that LJF can regulate the hepatocellular death and hepatocyte autophagy to protect the normal function of the liver in NAFLD. A total of 17 active components in LJF, and 29 targets related to NAFLD were predicted by network pharmacology. In total, 23 terms of biological process (BP), 20 entries of cellular component (CC), and 21 terms of molecular function (MF) were obtained by GO analysis, while 19 pathways associated with LJF against NAFLD were revealed by KEGG analysis. The molecular docking study of the main components and the key targets showed that isochlorogenic acid B can bind stably to TNF-α and CASP3. The in vitro studies have revealed that LJF extracts down-regulated TNF-α and CASP3 expression in an NAFLD cell model. Animal and clinical studies should be carried out to further confirm the mechanism of LJF.

## Figures and Tables

**Table 1:** Main active ingredients from LJF.

NO.	Name	Chemical Formula	2D Structure
H1	Neochlorogenic acid	C_16_H_18_O_9_	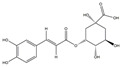
H2	Isochlorogenic acid B	C_25_H_24_O_12_	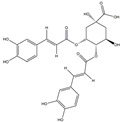
H3	Quercetin-O-glucoside	C_21_H_20_O_12_	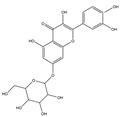
H4	Luteolin-O-glucoside	C_21_H_20_O_11_	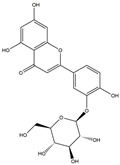
H5	Isorhamnetin-O-glucoside	C_22_H_20_O_12_	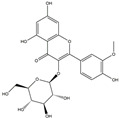
H6	Loganic acid	C_16_H_24_O_10_	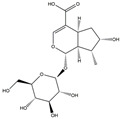
H7	Loganin	C_17_H_26_O_10_	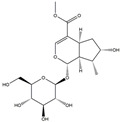
H8	Secoxyloganin	C_17_H_24_O_11_	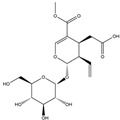
H9	Vogeloside	C_17_H_24_O_10_	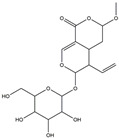
H10	Apigenin-glucuronide-sulfate	C_21_H_18_O_14_S	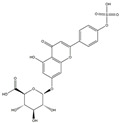
H11	Isorhamnetin-glucuronide-sulfate	C_22_H_22_O_16_S	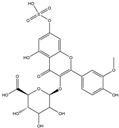
H12	Apigenin-glucuronide	C_21_H_18_O_11_	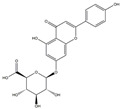
H13	5-Hydroxy-6-methoxyindole glucuronide	C_15_H_17_NO_8_	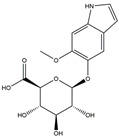
H14	Dihydrocaffeic acid-sulfate	C_9_H_10_O_7_S	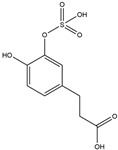
H15	Dihydroferulic acid-sulfate	C_10_H_12_O_7_S	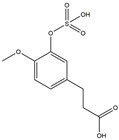
H16	Catechol sulfate	C_6_H_6_O_5_S	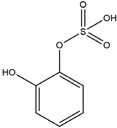
H17	Feruloylquinic acid	C_17_H_20_O_9_	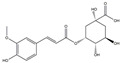

## Data Availability

Not applicable.

## Supplementary Materials

The following supporting information can be downloaded at: https://www.mdpi.com/article/10.3390/medicina58091176/s1, Figure S1: Typical chromatograms of mixed standards (A) and LJF samples (B); Table S1: Primer sequences of TNF-α and CASP3; Table S2: Protein function potential targets from main active ingredients of LJF against NAFLD; Table S3: Protein function potential targets from main active ingredients of LJF against NAFLD.
