# Supplementary material for: Investigation of Lonicera japonica Flos against Nonalcoholic Fatty Liver Disease Using Network Integration and Experimental Validation
Source: Medicina (Kaunas). 2022 Aug 30;58(9):1176. doi: 10.3390/medicina58091176 (PMC9506563; doi:10.3390/medicina58091176)
Supplement: Supplementary file 1 [file medicina-58-01176-s001.zip › medicina-1873759-supplementary.pdf]

Outline of the Supplementary material

Tables

Table S1 Primer sequences of TNF and CASP3

Table S2 Protein function potential targets from main active ingredients of LJF against NAFLD

Table S3 Protein function potential targets from main active ingredients of LJF against NAFLD

Figures

Figure S1. Typical chromatograms of mixed standards (A) and LJF samples (B).

Supplementary Table S1 Primer sequences of TNF and CASP3

Name	Sequences (5’-3’)
hGAPDH-F	GGAGCGAGATCCCTCCAAAAT
hGAPDH-R	GGCTGTTGTCATACTTCTCATGG
Hu-TNF-F	GAGGCCAAGCCCTGGTATG
Hu-TNF-R	CGGGCCGATTGATCTCAGC
Hu-CASP3-F	CATGGAAGCGAATCAATGGACT
Hu-CASP3-R	CTGTACCAGACCGAGATGTCA

Supplemental Table S2 Protein function potential targets from main active ingredients of LJF against NAFLD

NO.	Gene symbol	Description
1	APP	amyloid beta precursor protein
2	MMP2	matrix metallopeptidase 2
3	PYGL	glycogen phosphorylase L
4	PRKCD	protein kinase C delta
5	CA1	carbonic anhydrase 1
6	CASP3	caspase 3
7	ELANE	elastase, neutrophil expressed
8	CASP2	caspase 2
9	FTO	FTO alpha-ketoglutarate dependent dioxygenase
10	TTR	transthyretin
11	MMP1	matrix metallopeptidase 1
12	TNF	tumor necrosis factor
13	XDH	xanthine dehydrogenase
14	NOX4	NADPH oxidase 4
15	ACHE	acetylcholinesterase (Cartwright blood group)
16	RPS6KA3	ribosomal protein S6 kinase A3
17	PTGS2	prostaglandin-endoperoxide synthase 2
18	ALDH2	aldehyde dehydrogenase 2 family member
19	EGFR	epidermal growth factor receptor
20	TERT	telomerase reverse transcriptase
21	HSP90AA1	heat shock protein 90 alpha family class A member 1
22	ADORA2A	adenosine A2a receptor
23	IGFBP3	insulin like growth factor binding protein 3
24	ALOX12	arachidonate 12-lipoxygenase, 12S type
25	PABPC1	poly(A) binding protein cytoplasmic 1
26	HSPA5	heat shock protein family A (Hsp70) member 5
27	PTPN11	protein tyrosine phosphatase non-receptor type 11
28	FDFT1	farnesyl-diphosphate farnesyltransferase 1
29	PFKFB3	6-phosphofructo-2-kinase/fructose-2,6-biphosphatase 3

Supplemental Table S3 Protein function potential targets from main active ingredients of LJF against NAFLD

NO.	Gene symbol	Protein function
1	APP	amyloid beta precursor protein
2	MMP2	matrix metallopeptidase 2
3	PYGL	glycogen phosphorylase L
4	PRKCD	protein kinase C delta
5	CA1	carbonic anhydrase 1
6	CASP3	caspase 3
7	ELANE	elastase, neutrophil expressed
8	CASP2	caspase 2
9	FTO	FTO alpha-ketoglutarate dependent dioxygenase
10	TTR	transthyretin
11	MMP1	matrix metallopeptidase 1
12	TNF	tumor necrosis factor
13	XDH	xanthine dehydrogenase
14	NOX4	NADPH oxidase 4
15	ACHE	acetylcholinesterase (Cartwright blood group)
16	RPS6KA3	ribosomal protein S6 kinase A3
17	PTGS2	prostaglandin-endoperoxide synthase 2
18	ALDH2	aldehyde dehydrogenase 2 family member
19	EGFR	epidermal growth factor receptor
20	TERT	telomerase reverse transcriptase
21	HSP90AA1	heat shock protein 90 alpha family class A member 1
22	ADORA2A	adenosine A2a receptor
23	IGFBP3	insulin like growth factor binding protein 3
24	ALOX12	arachidonate 12-lipoxygenase, 12S type
25	PABPC1	poly(A) binding protein cytoplasmic 1
26	HSPA5	heat shock protein family A (Hsp70) member 5
27	PTPN11	protein tyrosine phosphatase non-receptor type 11
28	FDFT1	farnesyl-diphosphate farnesyltransferase 1
29	PFKFB3	6-phosphofructo-2-kinase/fructose-2,6-biphosphatase 3

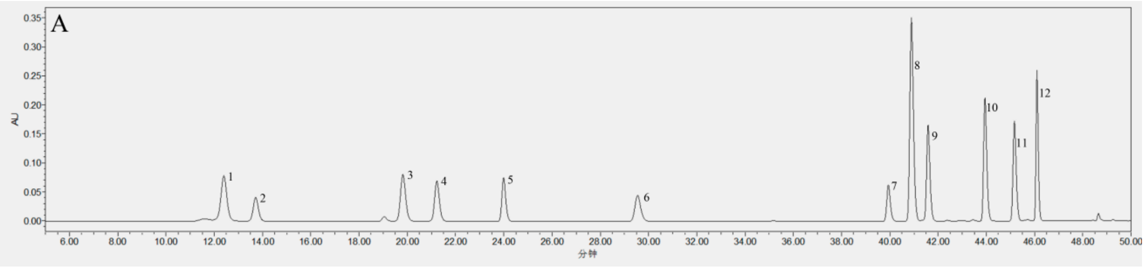

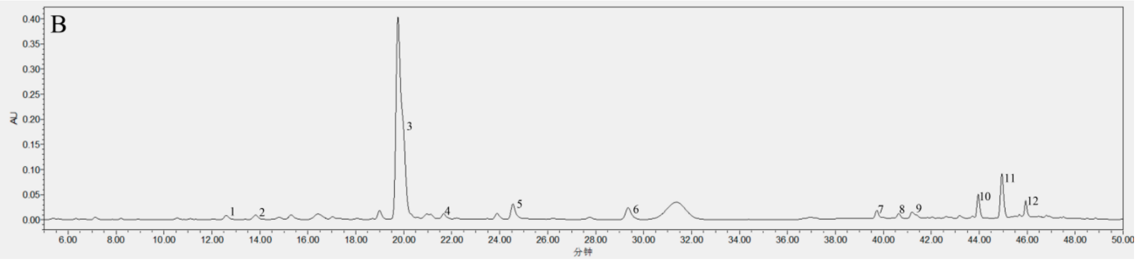

Supplemental Figure S1. Typical chromatograms of mixed standards (A) and LJF samples (B).

1. Neochlorogenic acid, 2. Loganic acid, 3. Chlorogenic acid, 4. Cryptochlorogenic acid, 5. Loganin, 6. Secoxyloganin, 7. Rutinum, 8. Hyperoside, 9. Isochlorogenic acid B, 10. Isochlorogenic acid A, 11. Isochlorogenic acid C, 12. Luteoloside.
